# Future directions of neoadjuvant therapy in pancreatic cancer

**DOI:** 10.18632/oncoscience.506

**Published:** 2020-05-14

**Authors:** Carmen Mota Reyes, Ümmügülsüm Yurteri, Helmut Friess, Ihsan Ekin Demir

**Affiliations:** ^1^Department of Surgery, Klinikum rechts der Isar, Technical University of Munich, School of Medicine, Munich, Germany; ^2^Department of General Surgery, HPB-Unit, School of Medicine, Acibadem Mehmet Ali Aydinlar University, Istanbul, Turkey; ^3^German Cancer Consortium (DKTK), Partner Site Munich, Munich, Germany; ^4^CRC 1321 Modelling and Targeting Pancreatic Cancer, Munich, Germany; ^*^These authors contributed equally.

**Keywords:** pancreatic cancer, neoadjuvant therapy, immunotherapy, tregs

## Abstract

Neoadjuvant therapy with conventional chemotherapies have visibly improved the prognosis of locally advanced pancreatic cancer (PCa). However, molecular targeted therapies that have provided durable responses in other tumor entities, have not yet found access into neoadjuvant therapy of PCa. In fact, due to the presence of the tumor burden serving as an antigen source for T cell priming, neoadjuvant chemotherapy may unleash a more potent antitumoral immune response than adjuvant or palliative chemotherapy.

Despite tremendous efforts, successful therapy of pancreatic cancer (PCa) remains a major challenge [1]. Finding the way to reprogram the dynamic tumor-promoting interactions in the tumor microenvironment will help ameliorate this dreadful disease via more effective and less-toxic means than classical chemotherapy (Figure 1) [2]. Over the past decade, neoadjuvant approaches with conventional chemotherapies have dramatically improved the prognosis of locally advanced tumors including PCa [3-5]. Indeed, up to 60% of patients with locally advanced PCa can become resectable upon response to neoadjuvant chemotherapy [6], and first results from neoadjuvant therapy in resectable PCa are promising [7]. So far, neoadjuvant therapy in PCa does not make use of molecular targeted therapy but rather of classical regimens such as FOLFIRINOX, with the exception of nab-paclitaxel that that depends on stromal albumin receptor availability. Molecular targeted therapies that have provided durable responses in other tumor entities, have not yielded the expected outcomes in PCa [8]. 

Cancer immunotherapy, led by immune checkpoint inhibitors (ICI) and cancer vaccines have shown notable long-term efficacy in many solid malignancies [9]. The limited success of immunotherapy on PCa is mainly due to the low tumor mutational burden and presence of an immunosuppresive tumor microenvironment (TME) enriched with myeloid derived suppressor cells (MDSC) and regulatory T cells (Tregs) with a paucity of cytotoxic T cells [9, 10]. On-going trials on PCa are now focusing on combinational therapies exploiting the ability of cancer vaccines to promote T cell recruitment, which are then consecutively activated by immune checkpoint inhibitors (ICIs) or immunomodulatory agents [10]. In murine models of PCa, the combination of an immune checkpoint agonist (OX40) and an anti-PD-1 antibody with a neoantigen-targeted vaccine resulted in tumor regression and increased survival [11]. Furthermore, the administration of chemotherapy prior to treatment with CD40 agonists may operate synergistically to prime antigen presenting cells by the release of tumor-associated antigens through cytotoxic cell death and thus enhance the response to these agents [10]. 

Adoptive immunotherapy involves injecting antitumor-programmed immune cells into the patient. New directions are pointing to tumor lysate-pulsed dendritic cells [5, 12] or the generation of cell lines expressing non autologous HLA-DR, which will trigger an immune response when administered to the patient [5]. The use of chimeric antigen receptor (CAR)-T cells reported promising results and procured Treg depletion in cell culture and mouse models [13]. Further advances, including engineering CAR-T cells to produce appropriate cytokines or suicide cassettes to limit toxicity, are in development [5]. 

The failure of immunotherapy in PCa may be partly due to the exclusion of T cells by cancer-associated fibroblasts (CAF) and the impaired drug delivery caused by increased hydrostatic pressure and the poor vascularization in the highly fibrotic pancreatic stroma [14]. Exploiting the stroma to improve T cell recruitment and chemotherapeutic drug delivery is therefore an attractive therapeutic target. Systemic administration of the modified hyaluronidase molecule PEGPH20 reduced tumoral hyaluronic content and vascular collapse in mice with PCa [10, 15]. This promising preclinical data led to clinical trials in which PEGPH20 is combined with ICIs that are currently recruiting patients. Targeting the fibrotic stroma is, however, at least controversial. A clinical trial of an inhibitor of the sonic Hedgehog signalling, a key regulator of tumor-stromal interactions, was stopped early because of an increased rate of progressive disease in the treatment arm [10, 14]. Therefore, it will be important to take into consideration the two-edged nature of the stroma in order to optimally target its components without compromising its protective role. 

While metabolic plasticity has long been recognized as a hallmark of cancer, we have only recently started to exploit the differences between cancer cell and normal cell metabolism [8]. Metabolic rewiring of PCa cells through oncogenic Kras permits the survival and proliferation in a severely hypoxic and nutrient-deprived microenvironment and presents assorted opportunities for selective targeting [3, 8]. Hydroxychloroquine has reached several clinical trials for PCa patients as a potent inhibitor of autophagy and micropynocitosis by preventing lysosomal degradation [8] showing clinical potential. Further key regulators of tumour metabolism such as pyruvate kinase isoform M2 and lactate transport also play a critical role in the maintenance of intracellular pH and in tumor-stroma interactions and are subjects of preclinical investigations [3]. Interestingly, recent studies demonstrated that fasting cycles enhanced the efficacy of gemcitabine *in vitro* and in the *in vivo* murine xenograft model and inhibited PCa growth [1]. 

Our growing understanding of the PCa biology has led to the development of novel immunotherapies as well as drugs targeting key regulators of the stromal and tumor metabolism. However, the full potential of these agents has been hampered by the presence of therapeutic resistance, resulting from intrinsic compensatory signalling pathways and mutagenic evolution [16]. Only combinations of targeted treatments addressing simultaneously the immunological and stromal component of the TME as well as tumor-promoting metabolic pathways, will be able to circumvent the manifold resistance mechanisms and yield a clinical benefit [16]. The main challenge will be to propose and predict effective combinations. On this matter, novel technologies such as gene-silencing tools and phosphoproteomics are crucial and will enable the identification of synthetic lethal drug combinations and complex compensatory signalling pathways in PCa cells [16]. Due to the presence of the tumor burden serving as an antigen source for T cell priming, neoadjuvant approaches may unleash a more potent antitumoral immune response [17] and seem undoubtedly the appropriate start for future multimodal strategies combining immune therapy with classical chemotherapy. 

**Figure 1:**
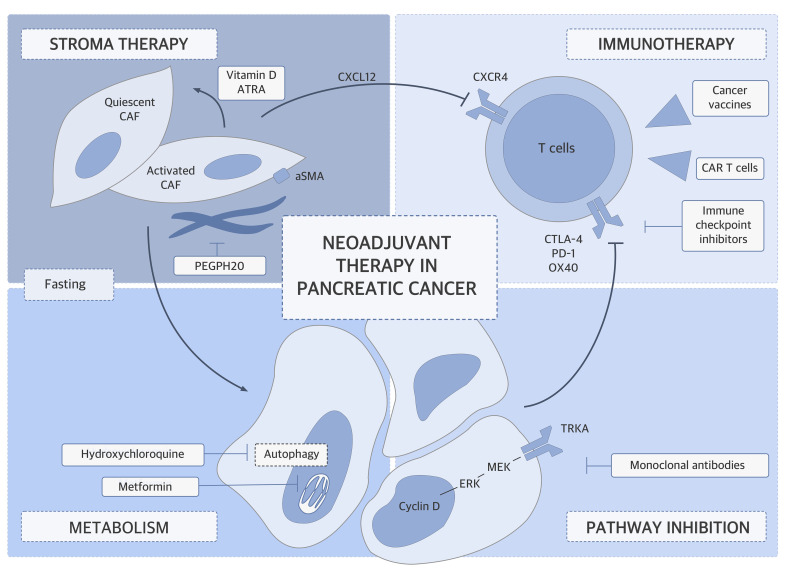
Neoadjuvant therapy at the crossroads of stroma, immunotherapy, and metabolism in pancreatic cancer. Future neoadjuvant therapy approaches should incorporate the recently discovered mechanisms in tumor biology for exploiting them toward tumor downsizing.
